# Vietnam Association of Gastroenterology (VNAGE) guideline on the management of gastroesophageal reflux disease

**DOI:** 10.3389/fmed.2026.1890726

**Published:** 2026-08-03

**Authors:** Hang Viet Dao, Bang Hong Mai, Long Van Dao, Huy Van Tran, Mien Kieu Tran, Duc Trong Quach, Khien Van Vu, Khanh Truong Vu, Dung Dang-Quy Ho, Trung Thien Tran, Ho Thi-Thu Pham, Dung Tuan Trinh, Vinh Thuy Nguyen, Hoan Quoc Phan, Duat Quang Nguyen, Hoang Huu Bui, Tuong Thi-Khanh Tran, Ha Thi-Viet Nguyen, Hong Thi-Van Nguyen, Thang Duy Nguyen, Binh Canh Nguyen, Thong Duy Vo, Ky Doan Thai, Nam Trung Phan, Nho Viet Le, Cong Hong-Minh Vo, Phat Tan Ho, Phuong Thi-Tuyet Le

**Affiliations:** 1 Department of Internal Medicine, Hanoi Medical University, Hanoi, Vietnam; 2 Institute of Gastroenterology and Hepatology, Hanoi, Vietnam; 3 Military Central Hospital, Hanoi, Vietnam; 4 Hue University of Medicine and Pharmacy, Hue, Vietnam; 5 Department of Internal Medicine, University of Medicine and Pharmacy at Ho Chi Minh City, Ho Chi Minh City, Vietnam; 6 Nhan Dan Gia Dinh Hospital, Ho Chi Minh City, Vietnam; 7 Tam Anh Hospital, Hanoi, Vietnam; 8 Cho Ray Hospital, Ho Chi Minh City, Vietnam; 9 Department of Internal Medicine, Hanoi National University, Hanoi, Vietnam; 10 Hanoi French Hospital, Hanoi, Vietnam; 11 Department of Internal Medicine, Pham Ngoc Thach University of Medicine, Ho Chi Minh City, Vietnam; 12 Department of Pediatrics, Hanoi Medical University, Hanoi, Vietnam; 13 Department of Internal Medicine, Da Nang University of Medical Technology and Pharmacy, Da Nang, Vietnam; 14 People’s Hospital 115, Ho Chi Minh City, Vietnam

**Keywords:** clinical practice guideline, gastroesophageal reflux disease, proton pump inhibitor, resource-limited settings, Vietnam

## Abstract

**Background and Aims:**

Gastroesophageal reflux disease (GERD) is common and heterogeneous, with growing recognition of nonerosive reflux disease (NERD) and extraesophageal manifestations in Asia. The variations in diagnostic resources and clinical practice necessitate context-specific, evidence-based guidance. Thus, we developed a guideline that aims to standardize the diagnosis and management of GERD in Vietnam.

**Methods:**

A multidisciplinary panel conducted a structured literature search and developed consensus statements using a modified Delphi process. Evidence quality and recommendation strength were evaluated using the GRADE framework.

**Results:**

Twenty recommendations address the diagnostic and therapeutic strategies. Upper endoscopy and ambulatory reflux monitoring are recommended for selected patients, whereas high-resolution manometry is advised for those with refractory symptoms and for preintervention evaluation. Empiric proton pump inhibitor (PPI) testing alone is insufficient for diagnosis. Lifestyle modification and optimized PPI therapy remain the first-line treatments. Standard-duration PPI therapy is recommended for patients with NERD and erosive esophagitis, with optimization required before defining refractory GERD. Potassium-competitive acid blockers may be considered as alternatives. Endoscopic and surgical interventions are reserved for patients with objective reflux and persistent symptoms despite undergoing optimized medical therapy.

**Conclusion:**

The developed guideline provides evidence-based, context-adapted recommendations to standardize GERD care in Vietnam.

## 1 Introduction

According to the Montreal Consensus, gastroesophageal reflux disease (GERD) is defined as a condition in which gastric contents move upward into the esophagus, leading to troublesome symptoms and/or complications. Recently, there has been an increasing prevalence of GERD symptoms across Southeast Asia (1–3). A systematic analysis of the global burden of GERD across 195 countries and territories from 1990 to 2017 revealed that the number of GERD cases in Southeast Asia has increased from over 29 million in 1990 to more than 52 million in 2017 (1). In Vietnam, a similar upward trend has been observed during the same period. This is the first consensus in Vietnam for the diagnosis and management of GERD. Over the past 5 years, regional and international guidelines have incorporated novel diagnostic modalities for the definitive and supportive diagnosis of GERD. However, the implementation of these diagnostic modalities for GERD in Vietnamese settings remains challenging due to its diverse presentations and limited healthcare capacity. Additionally, the therapeutic approach to GERD has not been standardized across regions, with treatment strategies varying based on the local resources, underlying pathophysiology, individual response to therapy, and coexistence of disorders of gut–brain interactions (DGBIs). Therefore, there is an urgent need for a unified and context-specific clinical guideline. Therefore, we aimed to develop a guideline that provides updated, evidence-based recommendations on the diagnosis and management of GERD in Vietnam. We also sought to outline the levels of evidence and strength of each recommendation to facilitate informed clinical decision-making.

## 2 Methods

The statements in this guideline, prepared by experts from the Vietnam Association of Gastroenterology (VNAGE), address key aspects of the diagnosis and treatment of GERD. Twenty-one draft statements with supporting evidence were distributed to all members of the Expert Panel. The guideline was developed using the Delphi consensus method.

The Expert Panel comprised 28 members, including experts in gastroenterology, pathology, gastrointestinal surgery, and endoscopy with expertise in GERD. Panelists assessed the level of evidence and strength of recommendation for each statement. The members were also invited to suggest additional key references that they deemed pertinent to the recommendation under review.

To develop each statement, a structured literature search and narrative synthesis of evidence were conducted in PubMed and other relevant Vietnamese sources, covering publications up to January 2025. For each statement, we formulated clinical questions in PICO format (Population, Intervention, Comparator, Outcome), provided in Supplementary Table 1 of the Supplementary Appendix. Evidence was selected based on its relevance to the clinical question, methodological design, clinical importance, and applicability to the Vietnamese healthcare context. Priority was given to systematic reviews, meta-analyses, randomized controlled trials, diagnostic accuracy studies, clinically relevant observational studies, as well as clinical practice guidelines and consensus statements from gastroenterology associations and societies. As this guideline was developed through an expert consensus process (Delphi process) rather than a formal systematic review, full risk-of-bias assessments and summary-of-findings tables were not produced.

Each statement was assessed using the Grading of Recommendations, Assessment, Development, and Evaluation (GRADE) system. The level of evidence was graded as high, moderate, or low, based on study design, consistency of findings, directness to the clinical question, precision of estimates, and applicability to the Vietnamese context. The strength of recommendations was graded as either strong or weak, based on the quality of evidence, the balance of benefits and risks, and feasibility in terms of cost and availability for application in the Vietnamese context. Therefore, a strong recommendation could be issued despite moderate or low certainty of evidence when the Expert Panel judged that the expected clinical benefit, feasibility, and contextual relevance outweighed the limitations of the available evidence. Regarding the level of consensus, each member would evaluate one of the following six levels: (1) accept completely; (2) accept with some reservations; (3) accept with major reservations; (4) reject with some reservations; (5) reject with major reservations; or (6) reject completely. A statement was approved if >80% of votes for consensus levels were within levels 1 or 2.

The voting process included three rounds. In the first round, all 28 panelists independently and anonymously voted on the 21 draft statements via an online platform. In the second round, all panelists participated in a hybrid meeting (in-person and virtual) to review and revise all the statements based on the first-round results. One statement regarding the treatment of *Helicobacter pylori* (*H. pylori*) infection in GERD patients was excluded due to substantial overlap with an existing *H. pylori* management guideline in Vietnam. The remaining 20 statements achieved ≥80% consensus, though several required updates to the supporting evidence. The Guidelines Committee compiled the consensus data, revised all statements, and added relevant new scientific evidence. In the third round, the revised statements were then submitted for final evaluation. All 20 statements were approved for inclusion in the final guideline.

## 3 Diagnosis of GERD

**Recommendation 1:** GERD presents various clinical symptoms, including esophageal and extraesophageal manifestations. Patients presenting solely with atypical symptoms should be evaluated through a multidisciplinary approach.

*Level of evidence: High*
*Recommendation level: Strong*
*Level of agreement: Strongly agree (92.9%); Agree with some concern (7.1%)*

GERD presents with a wide spectrum of symptoms, encompassing esophageal and extraesophageal manifestations. According to the Montreal consensus and the American College of Gastroenterology (ACG), the typical symptoms of GERD include heartburn and regurgitation. The Lyon Consensus 2.0 further recognized non-cardiac chest pain as the third typical symptom of GERD (2–4). Heartburn is characterized by a retrosternal burning sensation, often worsening after meals or in the supine position, whereas regurgitation refers to the reflux of gastric contents into the mouth. In Vietnam, the proportions of patients with heartburn and regurgitation in those undergoing esophagogastroduodenoscopy (EGD) for upper gastrointestinal (GI) symptoms were 9.2% and 27%, respectively. These figures were higher in patients with a presumptive GERD diagnosis, with prevalences ranging from 68.4% to 78.6% for heartburn and 71.5% to 77.4% for regurgitation (5–7). Besides the typical symptoms of GERD, the American Gastroenterological Association (AGA) advocates a multidisciplinary approach for evaluating atypical and extraesophageal symptoms (8). These manifestations account for 7%–23.1% of GERD presentations and mainly occur in various specialties besides gastroenterology, including respiratory medicine, dentistry, otorhinolaryngology, and cardiology (9, 10). The common atypical or extraesophageal symptoms include chronic cough, asthma, laryngitis, non-cardiac chest pain, and dental erosion (11). A previous study conducted in Vietnam investigated the prevalence of extraesophageal symptoms in patients with GERD. The three most common symptoms were coughing (49.1%), globus sensation (46.7%), and non-cardiac chest pain (45.3%). The less frequent symptoms were hoarseness (9.9%) and wheezing (9.4%) (7). Moreover, another study conducted in Vietnam reported a dental erosion rate of 78.1% in 169 GERD patients. Specifically, mild, moderate, and severe erosion were noted in 50%, 42%, and 8% of cases, respectively (12). The differential diagnosis of GERD from other causes in patients with extraesophageal manifestations requires careful evaluation and a multidisciplinary diagnostic approach.

**Recommendation 2:** Clinical questionnaires and proton pump inhibitor (PPI) trials are insufficient for establishing a definitive GERD diagnosis.

*Level of evidence: Moderate*
*Recommendation level: Strong*
*Level of agreement: Strongly agree (78.6%); Agree with some concern (14.3%); Agree with major concerns (7.1%)*

Clinical questionnaires, including the Gastroesophageal Reflux Disease Questionnaire (GerdQ) or Frequency Scale for Symptoms of GERD (FSSG), are simple, affordable, and non-invasive diagnostic tools for GERD, making them suitable for community-based screening. However, their diagnostic accuracy is limited. A GerdQ score of ≥8 has sensitivity and specificity values of 64.6% and 71.4%, respectively, for GERD diagnosis, whereas an FSSG score of ≥8 yields a sensitivity of 62% and a specificity of 59% (13, 14). In a Vietnamese cohort, the optimal cutoff score for GerdQ was lower than that of Western populations. A GerdQ score of ≥6 achieved a sensitivity of 70.3% and specificity of 72% (15). Furthermore, in another Vietnam study evaluating 24-h esophageal pH-impedance testing in 37 refractory GERD patients, the mean FSSG score was higher (19.1) and the mean GerdQ score was lower (8.29) in patients with abnormal esophageal acid exposure time (AET) than in those without abnormal AET, but the difference was not statistically significant (16).

Most American guidelines recommend a PPI trial for diagnosing GERD in patients with typical GERD symptoms or noncardiac chest pain because of the accessibility and affordability of PPIs (3, 17). However, the diagnostic accuracy of the PPI trial has been limited. A previous meta-analysis found that PPI therapy has a moderate sensitivity of 79% but a low specificity of 45% in differentiating GERD from non-GERD patients (18). The Diamond study involving 308 patients with upper GI symptoms noted that 35% of patients without GERD and 54% of those with confirmed GERD experienced symptom improvement after a 2-week PPI trial (19).

**Recommendation 3:** Esophagogastroduodenoscopy is recommended for patients with alarm symptoms, those with persistent reflux symptoms unresponsive to PPI trial, or those considered for endoscopic or surgical anti-reflux procedures.

*Level of evidence: High*
*Recommendation level: Strong*
*Level of agreement: Strongly agree (82.1%); Agree with some concern (17.9%)*

According to the ACG and American Society for Gastrointestinal Endoscopy, EGD is recommended for patients with alarm symptoms (dysphagia, odynophagia, persistent vomiting, unintentional weight loss, GI bleeding, or anemia), radiologic abnormalities of the upper GI tract (strictures, ulcerations, or tumors), risk factors for Barrett’s esophagus (age >50 years, reflux symptoms lasting for >5 years, a family history of esophageal cancer and/or Barrett’s esophagus, nocturnal reflux, hiatal hernia, overweight, or smoking) or before anti-reflux interventions (3, 20). In 2023, the VNAGE issued a guideline recommending EGD for women aged >35 years and men aged >40 years, based on the results of a large study involving 472,744 patients who underwent EGD, which reported a malignancy rate of 0.4%, with 6.5% of cases occurring in those aged <40 years (21, 22). In patients with extraesophageal symptoms, the ACG does not routinely recommend EGD, whereas the AGA suggests a comprehensive diagnostic approach of clinical symptoms, PPI trial, EGD, or esophageal pH monitoring (3, 8). A cross-sectional study found that GERD patients with extraesophageal symptoms have a higher rate of erosive esophagitis (EE) than those with typical symptoms (52.3% vs. 38.4%), although most cases were of mild grade (23). Another indication of EGD includes the presence of persistent or progressive symptoms refractory to standard-dose PPI treatment, as recommended by multiple societies, including the International Working Group for the Classification of Oesophagitis (IWGCO), European Society for Neurogastroenterology and Motility, and American Neurogastroenterology and Motility Society (ESNM/ANMS) (24, 25). Before EGD, ACG recommends discontinuing the PPI treatment for 2–4 weeks to optimize the diagnostic accuracy, as they may mask the mucosal lesions (3).

**Recommendation 4:** NERD is more prevalent and less responsive to PPI therapy compared to ERD. Severe complications of GERD, including hemorrhage, esophageal ulcers, strictures, or cancer, are relatively uncommon in Vietnam.

*Level of evidence: High*
*Recommendation level: Strong*
*Level of agreement: Strongly agree (67.9%); Agree with some concern (28.6%); Agree with major concerns (3.6%)*

**Table:** 

Summary of statements	Level of evidence	Strength of recommendation
**Diagnosis of gastroesophageal reflux disease (GERD)**		
1. GERD presents various clinical symptoms, including both esophageal and extraesophageal manifestations. Patients presenting solely with atypical symptoms should be evaluated through a multidisciplinary approach.	High	Strong
2. Clinical questionnaires and proton pump inhibitor (PPI) trials are insufficient for establishing a definitive GERD diagnosis.	Moderate	Strong
3. Esophagogastroduodenoscopy is recommended for patients with alarm symptoms, those with persistent reflux symptoms unresponsive to PPI trial, or those considered for endoscopic or surgical anti-reflux procedures.	High	Strong
4. Nonerosive reflux disease (NERD) is more prevalent and less responsive to PPI therapy compared to erosive reflux disease. Severe complications of GERD, including hemorrhage, esophageal ulcers, strictures, or cancer, are relatively uncommon in Vietnam.	High	Strong
5. 24-Hour esophageal pH monitoring, a definitive diagnostic method for GERD, is indicated in patients with persistent reflux symptoms, those non-responsive to PPI therapy, those with normal endoscopic findings or Los Angeles (LA) grade A esophagitis, or those with a scheduled endoscopic/surgical anti-reflux intervention.	High	Strong
6. Definite GERD is defined as the condition in which an individual meets at least one of the following criteria: (1) presence of typical reflux symptoms with endoscopic findings of LA grade B, C, and D esophagitis; biopsy-proven Barrett’s esophagus, or esophageal stricture; and (2) esophageal acid exposure time of >6% on 24-h pH monitoring.	High	Strong
7. High-resolution esophageal manometry is recommended for patients with persistent reflux symptoms unresponsive to treatment or symptoms suggestive of esophageal motility disorders, and for preoperative evaluation prior to endoscopic or surgical antireflux procedures.	High	Strong
8. Some other tests, including rapid salivary pepsin detection or mucosal impedance measurement, have been proposed as supportive tools for the diagnosis of GERD; however, more clinical data are needed.	Moderate	Weak
9. A multidisciplinary approach is recommended for patients presenting with extraesophageal symptoms in the absence of typical reflux symptoms to establish a conclusive GERD diagnosis before initiating a PPI trial.	Moderate	Strong
10. Refractory GERD is defined as the persistence of symptoms despite optimized medical therapy in patients with objective evidence of a GERD diagnosis.	High	Strong
**Treatment of GERD**		
11. The treatment of GERD, including dietary and lifestyle modifications in combination with optimized medical therapy, should be individualized. Endoscopic or surgical anti-reflux procedures should only be considered in appropriately selected cases.	High	Strong
12. Diet and lifestyle modifications, including weight loss and elevating the head of the bed while sleeping, are recommended to alleviate reflux symptoms.	Moderate	Strong
13. PPIs are the cornerstone of GERD treatment. An 8-week course of standard-dose PPI therapy is effective in promoting esophageal mucosal healing and symptom relief in patients with erosive esophagitis confirmed on endoscopy.	High	Strong
14. Patients with mild-to-moderate GERD are recommended to start with dietary and lifestyle modifications, followed by alginate therapy for symptom relief. If symptoms persist, treatment with PPIs alone or in combination with alginates should be considered.	Moderate	Strong
15. A standard-dose PPI therapy for 4–8 weeks is recommended for NERD.	High	Strong
16. PPI therapy is recommended for patients with extraesophageal presentations accompanied by typical reflux symptoms. Contrarily, patients with extraesophageal symptoms in the absence of typical GERD symptoms should undergo further diagnostic functional testing to confirm GERD before PPI treatment initiation.	Moderate	Strong
17. For patients with refractory GERD, it is necessary to evaluate the optimization of medical treatment, including lifestyle modifications and assessment of patient adherence, before considering dose escalation, switching to another PPI, or adding adjunctive therapies.	High	Strong
18. Potassium-competitive acid blockers could be considered for controlling acid secretion in GERD management.	Moderate	Strong
19. Endoscopic or surgical anti-reflux procedures may be considered for selected patients who have objective evidence of GERD and continue to experience troublesome typical reflux symptoms despite receiving optimized medical therapy.	Low	Strong
**Overlapping treatment of GERD and other functional disorders**		
20. In patients with GERD overlapping with other disorders of gut–brain interaction, treatment should include a combination of acid suppression and adjunctive pharmacologic therapies.	Moderate	Strong

GERD can be classified into the following three subgroups: erosive reflux disease (ERD), NERD, and Barrett’s esophagus (4). NERD is the predominant type, ranging from 50% to 85% overall and 78% to 93% in the Asia-Pacific region (26, 27). Studies in major hospitals in Vietnam have reported variable prevalence of NERD, ranging from 19.6% to 62.6% (28–30). NERD patients exhibit a lower response rate to PPI treatment than ERD patients (31). The reduced responsiveness of NERD to PPI therapy may be attributable to conditions such as non-acid reflux, functional heartburn, and esophageal hypersensitivity. A systematic review of seven clinical trials reported that the response rate to 4-week PPI therapy was significantly lower in patients with NERD than in patients with ERD (37% vs. 56%, p < 0.001) (32). Establishing a conclusive diagnosis of NERD requires esophageal 24-h pH monitoring with or without impedance. Esophageal biopsy should also be considered in patients with suspected eosinophilic esophagitis (EoE), particularly those presenting with dysphagia, obstruction, or inadequate response to PPI therapy.

Serious complications of GERD include GI bleeding, esophageal ulcers, and esophageal strictures. These complications can reach up to 10% and 7% for esophageal strictures and ulcers, respectively (33, 34). The rates are relatively lower in the Asian population, ranging from 0.08% to 4.8% (35–37). The esophageal ulcer and bleeding rates were low in Vietnam at 1.3%–5% and 0.4%, respectively (7, 28).

**Recommendation 5:** 24-Hour esophageal pH monitoring, a definitive diagnostic method for GERD, is indicated in patients with persistent reflux symptoms, those nonresponsive to PPI therapy, those with normal endoscopic findings or Los Angeles grade A esophagitis, or those with scheduled endoscopic/surgical anti-reflux intervention.

*Level of evidence: High*
*Recommendation level: Strong*
*Level of agreement: Strongly agree (75%); Agree with some concern (21.4%); Agree with major concerns (3.6%)*

Esophageal pH monitoring is a diagnostic method for GERD based on AET. This test can be performed either on or off PPI therapy. The 2024 IWGCO consensus recommended pH monitoring off-PPI for patients with suspected refractory GERD who lack prior objective evidence of GERD, or pH testing on-PPI for those with persistent symptoms to identify the presence of acid or non-acid reflux (24). According to the 2022 ACG guideline and Lyon consensus 2.0, this test should be indicated after discontinuing the PPI therapy for at least 7 days in patients with persistent reflux symptoms and no objective evidence of GERD, or in those being considered for invasive anti-reflux procedures (3, 4). Recent advances include wireless pH monitoring, which offers extended monitoring times of up to 96 h. The Lyon Consensus 2.0 and 2022 AGA guideline support extended monitoring durations to optimize the diagnostic yield (3, 8). In a previous study involving 132 PPI-refractory patients, the accuracy of wireless pH monitoring at 72–96 h in predicting PPI treatment discontinuation was significantly higher than that at 48 h (area under the receiver operating characteristic (ROC) curve (AUC)) value, 0.63 vs. AUC, 0.57; *p* = 0.01) (38). However, wireless pH monitoring remains unavailable in many medical facilities, particularly in resource-limited regions (4).

Despite its diagnostic utility, the Southeast Asia (SEA) consensus does not recommend the routine use of 24-h pH monitoring in the region due to limited accessibility and the high prevalence of mild-to-moderate GERD (39). Therefore, in the context of Vietnam, esophageal pH monitoring should be reserved for selected cases and restricted to specialized medical facilities to avoid unnecessary healthcare expenditures.

**Recommendation 6:** Definite GERD is defined as the condition in which an individual meets at least one of the following criteria: (1) presence of typical reflux symptoms with endoscopic findings, such as LA grade B, C, D esophagitis, biopsy-proven Barrett’s esophagus, or esophageal stricture; and (2) esophageal AET of >6% on 24-h pH monitoring.

*Level of evidence: High*
*Recommendation level: Strong*
*Level of agreement: Strongly agree (92.9%); Agree with some concern (7.1%)*

Among the available diagnostic tools, EGD and 24-h esophageal pH monitoring are the two primary modalities for GERD confirmation. According to the Lyon consensus 1.0 (2018), a definitive diagnosis of GERD via EGD is established in patients presenting severe EE (LA grade C or D), biopsy-proven Barrett’s esophagus, or esophageal stricture (40). The diagnostic criteria for GERD via EGD have been expanded to include LA grade B esophagitis by the 2022 ACG guidelines and Lyon consensus 2.0 (3, 4). Performing EGD off-PPI therapy is recommended to optimize the detection of reflux-related mucosal lesions, as PPIs promote mucosal healing and obscure endoscopic findings (3, 41–43). 24-Hour esophageal pH monitoring, with or without impedance, provides objective evidence of reflux by quantifying esophageal acid exposure and characterizing the reflux events. These data are essential in diagnosing GERD and optimizing treatment. According to the 2017 Porto and Lyon consensus, an AET of >6% serves as the threshold for a conclusive diagnosis of GERD using either catheter-based or wireless pH monitoring (4, 40, 44). The upper limit of AET was lower in Asian populations than in European populations (3.2% vs. 8.2%) (45). Therefore, an AET of ≥4% can be considered an abnormal threshold in Asian populations, including Vietnam, although this threshold may not be sufficient to cause reflux symptoms (46, 47). However, diagnostic data defining the optimal AET cutoff for GERD diagnosis in Asian populations, particularly in Vietnam, remain limited. Therefore, this guideline retains AET >6% as a conservative criterion for definite GERD, in line with international consensus recommendations, to improve diagnostic specificity and minimize overdiagnosis. In patients with borderline AET values, clinical interpretation should incorporate reflux symptoms, endoscopic findings, reflux episodes, symptom association, and adjunctive impedance metrics rather than relying on AET alone. Additionally, some supportive evidence of GERD diagnosis on 24-h pH impedance monitoring includes the number of total reflux events, mean nocturnal baseline impedance (MNBI), post-reflux swallow-induced peristaltic wave (PSPW), and indices of symptom–reflux correlations (4). In a Vietnamese study on 149 patients with reflux symptoms and healthy controls, GERD patients had significantly lower PSPW and MNBI than non-GERD patients (24.43% and 1001.72Ω vs. 34.76% and 2331Ω, respectively, *p* < 0.05) (48). Another Vietnamese cross-sectional study further evaluated the diagnostic value of the PSPW index in 112 patients with suspected reflux symptoms undergoing 24-h pH-impedance monitoring. The PSPW index differed significantly between GERD and non-GERD patients and correlated with the DeMeester score, MNBI, AET, and the number of acid reflux episodes. The PSPW index showed moderate diagnostic accuracy for GERD, with an AUC of 0.72, sensitivity of 50%, specificity of 83.3%, and an optimal cutoff value of 22% (49). According to the Lyon Consensus 2.0, MNBI of <1500 Ω is considered supportive evidence of GERD, whereas MNBI of >2500 Ω is valuable in excluding the diagnosis of GERD (4). A recent observational study in Vietnam provided additional support for using MNBI as an adjunctive impedance metric in GERD diagnosis. A lower MNBI threshold around 1500 Ω improved diagnostic specificity compared with the previously used threshold of 2292 Ω, whereas higher MNBI values remained useful for excluding GERD (50). These findings support the context-specific application of MNBI in Vietnamese patients, while also highlighting the need for further normative data from healthy Vietnamese populations. The assessment of the association between symptoms and reflux events using the Symptom Index (SI) and Symptom Association Probability (SAP) allows for differentiating GERD from esophageal hypersensitivity (normal AET with positive symptom association) and functional heartburn (normal AET without symptom association) on 24-h pH-impedance monitoring (44).

**Recommendation 7:** High-resolution esophageal manometry is recommended for patients with persistent reflux symptoms unresponsive to treatment or symptoms suggestive of esophageal motility disorders, and for preoperative evaluation prior to endoscopic or surgical anti-reflux procedures.

*Level of evidence: High*
*Recommendation level: Strong*
*Level of agreement: Strongly agree (82.1%); Agree with some concern (14.3%); Agree with major concerns (3.6%)*

The 2020 ESNM/ANMS and 2024 IWGCO consensus recommend high-resolution esophageal manometry (HRM) for patients with persistent reflux symptoms to rule out severe esophageal motility disorders and detect abnormalities in the esophagogastric junction (EGJ) and esophageal motility associated with GERD (24, 25). The 2015 World Gastroenterological Organization and 2022 ACG guidelines also recommend HRM to locate the lower esophageal sphincter (LES) prior to the 24-h pH monitoring or to evaluate the esophageal motility disorders, especially achalasia, before endoscopic or surgical anti-reflux interventions (3, 51). Achalasia, characterized by abnormal relaxation of the LES along with loss of normal esophageal motility, may present with symptoms overlapping GERD, with the reported rates of heartburn and regurgitation reaching up to 68% and 75.7%, respectively (52–55).

According to the Lyon Consensus 2.0, HRM provides evidence supporting the diagnosis of GERD, including reduced EGJ pressure, hiatal hernia, and esophageal hypomotility (4). Additionally, high-resolution impedance manometry is employed to assess abnormalities of the anti-reflux barrier, including transient lower esophageal sphincter relaxations (TLESRs), low LES pressure, and impaired esophageal clearance. A Vietnamese study involving 217 patients with reflux symptoms found that 46.5% of them had esophageal hypomotility, and LES pressure was significantly lower in these patients than in those with normal motility (56). Previous studies conducted in patients with absent contractility in Vietnam showed that the LES pressure and integrated relaxation pressure in 4 s (IRP4s) were low in patients with esophagitis. A low IRP4 increased the risk of developing EE by 2.21 times (57, 58). Another Vietnamese study using Chicago classification 3.0 has observed that patients with severe esophageal hypomotility (>70% ineffective swallows) and absent contractility had significantly higher prevalence of EE LA grades B–D (4.4% and 7%, respectively) than those with normal esophageal motility (2.6%). A similar trend was seen with short-segment Barrett’s esophagus, with prevalence rates of 5.6% and 6.3% in the respective groups, compared to 3.5% in the normal motility group (59). A large retrospective study applying Chicago Classification 4.0 in 2,219 patients with upper gastrointestinal symptoms reported that 42.2% had esophageal motility disorders on HRM, with ineffective esophageal motility being the most frequent abnormality. The proportion of ineffective esophageal motility tended to be higher among patients with GERD than among the overall upper gastrointestinal symptom population (60). Another Vietnamese study evaluating transient lower esophageal sphincter relaxations (TLESRs) in 100 patients with reflux symptoms found that 44% had TLESRs on HRM, and 60.8% of TLESR events were associated with reflux (61). These findings suggest that HRM may provide clinically relevant information on esophageal hypomotility, EGJ dysfunction, and reflux mechanisms in Vietnamese patients, particularly in specialized centers where esophageal function testing is available.

**Recommendation 8:** Some other tests, including rapid salivary pepsin detection or mucosal impedance measurement, have been proposed as supportive tools for the diagnosis of GERD; however, more clinical data are needed.

*Level of evidence: Moderate*
*Recommendation level: Weak*
*Level of agreement: Strongly agree (50%); Agree with some concern (35.7%); Agree with major concerns (14.3%)*

Non-invasive methods have been developed to support the diagnosis of GERD, as definitive diagnostic modalities (EGD, pH monitoring) may be invasive, costly, or not readily available in Southeast Asia. Several systematic reviews and meta-analyses have shown moderate diagnostic accuracy of GERD for salivary pepsin detection, with pooled specificity of approximately 62%–71%, pooled sensitivity of 60%–74%, and AUC value of ∼ 0.7 (62). In Vietnam, a cross-sectional study (99 patients with extraesophageal reflux symptoms and 50 healthy controls) found a higher prevalence of positive salivary pepsin in patients with GERD, but the diagnostic thresholds yielded low specificity for differentiation from non-GERD and healthy individuals (48). Endoscopy-guided esophageal mucosal integrity evaluation via mucosal impedance (MI) or mucosal admittance (MA) may support the diagnosis of GERD (63). Distinct MI profiles may differentiate GERD, EoE, and non-GERD conditions, highlighting MI’s potential for differential diagnosis (64–66). As for MA, the inverse of MI, a cross-sectional study (33 patients with PPI-resistant heartburn) found significantly higher MA in patients with EE than those with functional heartburn (67). In Vietnam, a cross-sectional study (92 patients) reported that an MA-integrated model had moderate accuracy (AUC value of 0.704) and a cutoff value of 45.2 measured at 5 cm above the Z-line yielded moderate sensitivity and specificity values of 74% and 78%, respectively (30).

**Recommendation 9:** A multidisciplinary approach is recommended for patients presenting with extraesophageal symptoms in the absence of typical reflux symptoms to establish a conclusive GERD diagnosis before initiating a PPI trial.

*Level of evidence: Moderate*
*Recommendation level: Strong*
*Level of agreement*: *Strongly agree (67.9%); Agree with some concern (25%); Agree with major concerns (7.1%)*

Experts from the AGA suggest a multidisciplinary approach for both GERD diagnosis and management with extraesophageal manifestations, involving various specialties such as otorhinolaryngology, respiratory medicine, dentistry, and cardiology (8). These manifestations occur in 7%–23.1% of patients with GERD and may present as laryngopharyngeal diseases (e.g., wheezing, hoarseness, chronic cough, laryngitis), respiratory disorders (e.g., asthma, pulmonary fibrosis), or other conditions such as sinusitis, dental erosion, and otitis media (9, 10). These symptoms may be reflux-related or due to non-GERD disorders, making the diagnosis challenging, particularly in the absence of typical GERD symptoms. The 2023 International Federation of Oto-Rhino-Laryngological Societies and 2025 San Diego consensus address diagnostic approaches to laryngopharyngeal reflux disease (LPRD), including clinical assessment, laryngoscopy, HRM, and hypopharyngeal–esophageal pH-impedance monitoring. LPRD is diagnosed when bothersome laryngopharyngeal symptoms coexist with objective evidence linking symptoms to reflux (68, 69). Laryngoscopy may show reflux-related findings, but they have low specificity and are common in asymptomatic individuals (70, 71). Pathologic GERD is confirmed by 24-h pH or pH-impedance monitoring in only 12.2%–30.3% of suspected LPR cases, limiting the diagnostic value of laryngoscopy and mainly serving to exclude non-reflux causes, such as malignancy (48, 72). The role of EGD remains unclear; although EE is more frequent in patients with extraesophageal symptoms than in those with typical GERD (52.3% vs. 38.4%, p < 0.05) (73), it does not establish causality. EGD is recommended in patients who fail PPI therapy or present with alarm features (68).

Esophageal pH monitoring is advised before PPI therapy in suspected extraesophageal GERD, particularly in cases without typical symptoms, but its utility in LPR diagnosis is limited. Hypopharyngeal–esophageal pH-impedance monitoring may help evaluate LPR, although further validation is needed (68).

**Recommendation 10:** Refractory GERD is defined as the persistence of symptoms despite optimized medical therapy in patients with objective evidence of a GERD diagnosis.

*Level of evidence: High*
*Recommendation level: Strong*
*Level of consensus*: *Strongly agree (82.1%); Agree with some concern (7.1%); Agree with major concerns (3.6%); Strongly disagree (7.1%)*

The diagnostic criteria for refractory GERD vary across regional guidelines. The ACG or AGA guidelines define refractory GERD as the presence of persistent symptoms despite treatment with double-dose PPI for at least 8 weeks (74). The Asia-Pacific consensus defines it as persistent symptoms following at least 8 weeks of standard-dose PPI treatment (75). The 2020 ESNM/ANMS consensus proposes a unified definition of refractory GERD and distinguishes refractory GERD, persistent reflux symptoms, and persistent reflux-like symptoms based on objective GERD evidence, testing context (on/off PPI), and response to optimized PPI therapy (25). For this guideline, optimized medical therapy refers to optimization of acid-suppressive therapy, including confirmation of treatment adherence and appropriate PPI use, dose escalation or switching to a more potent PPI (PCAB is not mentioned because it is not available in Vietnam), and adjunctive therapy when indicated (e.g., alginates, H2RAs, baclofen, or prokinetics).

In Asia, persistent symptoms despite receiving PPI therapy have been reported in 37.9% of NERD patients after 6 months in China, 16.7% of NERD and 6.6% of ERD patients after 8 weeks in Korea, and approximately 20%–30% of GERD patients – predominantly NERD – in Japan (75). A Vietnamese report involving 101 physicians showed that 88% of the cases had treatment-refractory GERD and 71.7% reported non-response rates of ≥ 10% in practice; however, the study lacked rigorous diagnostic criteria and Asia-Pacific-based quantitative data (52). Patients with persistent reflux symptoms after optimizing lifestyle changes and medical treatment require thorough investigations to confirm a refractory GERD diagnosis. In Vietnam, data on refractory GERD are scarce, leading to a lack of standardized national protocols for the assessment or monitoring of these patients.

## 4 Treatment of GERD

**Recommendation 11:** The treatment of GERD, including dietary and lifestyle modifications in combination with optimized medical therapy, should be individualized. Endoscopic or surgical anti-reflux procedures should only be considered in appropriately selected cases.

*Level of evidence: High*
*Recommendation level: Strong*
*Level of consensus: Strongly agree (92.9%); Agree with some concerns (7.1%)*

Management of GERD requires an integrated approach based on symptoms, endoscopic findings, and pathophysiology, aiming to relieve symptoms, heal mucosal injury, prevent the development of recurrence and complications, and improve the patients’ quality of life (3, 39, 43, 51). The treatment with GERD includes lifestyle and dietary modifications, optimized acid suppression, and endoscopic or surgical interventions in selected cases. The key non-pharmacological measures include weight loss, smoking and alcohol cessation, dietary changes, and head-of-bed elevation for nocturnal symptoms (39).

PPIs remain the cornerstone of GERD treatment. However, it needs to be individualized and optimized to avoid unnecessary long-term overuse (76). The initial dose and duration of PPI therapy should depend on the frequency and severity of clinical symptoms, extraesophageal manifestations, endoscopic findings, as well as prior treatment response (3, 17, 39). The combination of PPIs with other medications, including antacids, alginates, prokinetics, or neuromodulators, should be guided by clinical symptoms, pathogenesis, presence of overlapping DGBIs, and comorbidities (3, 25, 39, 77). Endoscopic or surgical anti-reflux interventions should only be considered in patients with a confirmed GERD diagnosis and typical reflux symptoms that are unresponsive to optimized medical treatments or those who are intolerant to medication (25). Careful reassessment and functional testing are essential before performing any intervention to avoid unnecessary invasive procedures. Fundoplication is effective, whereas newer endoscopic techniques (e.g., anti-reflux mucosectomy [ARMS] or transoral incisionless fundoplication [TIF]) are promising but lack long-term evidence in Vietnam (78–80).

**Recommendation 12:** Diet and lifestyle modifications, including weight loss and elevating the head of the bed while sleeping, are recommended to alleviate reflux symptoms.

*Level of evidence: Moderate*
*Recommendation level: Strong*
*Level of agreement: Strongly agree (71.4%); Agree with some concern (21.4%); Agree with major concerns (7.1%)*

Weight gain, smoking, alcohol use, and diet are recognized as risk factors for GERD; therefore, lifestyle and dietary modifications can alleviate the symptoms (81, 82).

***Weight management***

Obesity is strongly associated with GERD and its complications (83–85). Weight loss improves the symptoms only in obese patients; in the HUNT cohort (*n* = 29,610), reduction of the body mass index of ≥3.5 was linked to a 40% symptom reduction (86, 87). In two randomized controlled trials, weight loss also reduced the AET from 5.6% to 3.7% and from 8% to 5.5%, supporting strong recommendations for weight reduction in obese GERD patients (88).

***Elevating the head of the bed***

Given that reflux is more frequent supine, elevating the head of the bed is recommended for nocturnal symptoms (81). Elevating the head of the bed by 20 cm reduced acid exposure in a study of 204 patients (85, 89) and significantly improved nocturnal reflux in a randomized trial of 39 patients (RR: 2.08, 95% confidence interval [CI]: 1.19–3.61) (90).

***Smoking cessation***

Smoking is a recognized risk factor for GERD (81). In the previous HUNT study involving 29,610 patients, smoking cessation significantly alleviated the severe reflux symptoms in normal-weight patients receiving anti-reflux therapy at least once a week (odds ratio: 5.67, 95% CI: 1.36–23.64), but not in overweight or obese individuals (91). Another study also showed greater improvement in symptoms after 1 year (43.9% vs. 18.2%, *p* < 0.05) (92).

***Diet***

A survey involving 4,400 Vietnamese individuals identified overeating, tight clothing, stress, insomnia, and consumption of greasy foods, sour/spicy soups, citrus fruits, carbonated beverages, beer, and orange juice as factors associated with reflux symptoms (93), although evidence for specific foods remains inconsistent (94–99). Short meal intervals (<3 h) and a short meal-to-bed time are associated with increased symptoms and greater medication use (88).

**Recommendation 13:** PPIs are the cornerstone of GERD treatment. An 8-week course of standard-dose PPI therapy is effective in promoting esophageal mucosal healing and symptom relief in patients with erosive esophagitis confirmed on endoscopy.

*Level of evidence: High*
*Recommendation level: Strong*
*Level of agreement: Strongly agree (100%)*

Consistent evidence and international guidelines support the use of standard-dose PPIs as the first-line therapy for GERD, particularly ERD, due to their superior efficacy in mucosal healing and symptom control (3, 43). A meta-analysis (43 studies, 7,635 patients with ERD on PPI monotherapy) demonstrated esophageal mucosal healing rates of 65%, 80%, and 84% at 4, 8, and 12 weeks, respectively (100). Mild EE typically heals rapidly, making a 4-week treatment period potentially sufficient, especially in Asian populations where severe EE (LA grade C or D) is less prevalent as compared to Western populations (101). Two additional meta-analyses confirmed that standard-dose PPIs were more effective than H2-receptor antagonists in healing EE of all grades (RR 0.51; 95% CI: 0.44–0.59) and doubling the PPI dose may be considered for refractory symptoms or EE, although the economic burden should be weighed before dose escalation (102, 103).

International guidelines recommend performing repeat EGD after 6–8 weeks of PPI treatment only in patients with severe EE (LA grade C or D) to assess healing status and complications (esophageal stricture, Barrett’s esophagus, or esophageal cancer), as severe EE can obscure other mucosal lesions (104–106). A study involving 964 patients with severe EE undergoing repeat endoscopy has reported that the detection rates of new histological findings, esophageal stricture, dysplasia, esophageal cancer, and Barrett’s esophagus were 13.2%, 2.3%, 0.5%, 0.3%, and 10.1%, respectively (107).

**Recommendation 14:** Patients with mild-to-moderate GERD are recommended to start with dietary and lifestyle modifications, followed by alginate therapy for symptom relief. If symptoms persist, treatment with PPIs alone or in combination with alginates should be considered.

*Level of evidence: Moderate*
*Recommendation level: Strong*
*Level of agreement: Strongly agree (75%); Agree with some concern (25%)*

Although the prevalence of GERD is increasing in Asia, most patients present with mild-to-moderate GERD (108). According to the 2021 SEA consensus, GERD severity is defined by symptom burden, with mild GERD characterized by easily tolerated symptoms and moderate GERD by bothersome symptoms affecting daily activities; lifestyle modification is recommended as the first-line therapy for all patients (39).

Given the role of postprandial acid pockets in the pathophysiology of GERD (109), alginates, which form a barrier preventing reflux into the esophagus, are recommended as a next-step treatment for mild-to-moderate GERD when non-pharmacological measures are insufficient (110, 111). A key advantage of alginates is their rapid onset of action, typically within 1 h, which is faster than PPIs and H2-receptor antagonists (112). The recommendations for the treatment of mild-to-moderate GERD according to the SEA consensus (39).

A systematic review and meta-analysis (14 studies; 2,095 patients) has shown that alginate-containing regimens were more effective than placebo or antacids for symptom relief (OR 4.42; 95% CI: 2.45–7.97), with no statistically significant difference compared with PPIs or H2-receptor antagonists (OR 0.58; 95% CI: 0.27–1.22) (113). Moreover, randomized clinical trials showed that alginates were non-inferior to omeprazole for early heartburn relief in moderate GERD (114) and that alginate–PPI combination therapy improved the heartburn-free rates compared with PPI monotherapy in NERD patients (7 days: 56.7% vs. 25.7%; 28 days: 54.9% vs. 35.9%; *p* < 0.05) (115).

**Recommendation 15:** A standard-dose PPI therapy for 4–8 weeks is recommended for NERD.

*Level of evidence: High*
*Recommendation level: Strong*
*Level of agreement: Strongly agree (82.1%); Agree with some concern (17.9%)*

GERD without esophagitis confirmed on endoscopy (NERD) accounts for the majority of GERD cases (26), with a reported prevalence of 78%–93% in the Asia–Pacific region (27, 75, 108) and approximately 50% among Vietnamese patients with reflux symptoms (5, 116). PPIs are more effective than H2-receptor antagonists for symptom relief in patients with NERD (32, 117–120) and are recommended for a minimum treatment duration of 4 weeks based on the available clinical trial data (32, 121). However, the therapeutic response to PPIs in NERD appears less favorable as compared to ERD patients (122). Symptom control is achieved in only 30%–35% of NERD patients. The response rate after 4 weeks of treatment was significantly lower in patients with NERD than in those with ERD (37% vs. 56%, *p* < 0.0001) (118). These findings suggest that a 4-week treatment course may be insufficient to adequately assess the therapeutic efficacy of PPIs in this patient population (32).

After symptom resolution, on-demand PPI therapy has been reported as an effective strategy for managing symptom recurrence in NERD among several aspects, including treatment efficacy, patient satisfaction, tolerability, and cost-effectiveness (123–125). Previous meta-analyses have demonstrated that on-demand PPIs were superior to placebo and daily PPI therapy, with lower early treatment discontinuation rates (vs. placebo: 12.3% vs. 39.8%; OR: 0.22; 95% CI: 0.13–0.36; vs. daily PPI: 5.7% vs. 12.1%; OR: 0.44; 95% CI: 0.29–0.66), and were comparable to continuous PPI therapy in terms of symptom control, treatment failure, and patient satisfaction, particularly in NERD and mild ERD patients and Asian populations (124, 125).

**Recommendation 16:** PPI therapy is recommended for patients with extraesophageal presentations accompanied by typical reflux symptoms. Contrarily, patients with extraesophageal symptoms in the absence of typical GERD symptoms should undergo further diagnostic functional testing to confirm GERD before PPI treatment initiation.

*Level of evidence: Moderate*
*Recommendation level: Strong*
*Level of agreement: Strongly agree (78.6%); Agree with some concern (17.9%); Agree with major concerns (3.6%)*

Up to 50%–60% of patients with suspected extraesophageal reflux symptoms do not have a definitive diagnosis of GERD and fail to respond to acid suppression therapy (126, 127). In such cases, esophageal or oropharyngeal–esophageal pH monitoring is recommended to confirm the GERD diagnosis before starting PPI treatment (3). Although PPIs are the mainstay of GERD treatment, their efficacy in alleviating extraesophageal symptoms remains controversial. A pooled analysis of eight clinical trials indicated no significant benefit of PPI treatment over placebo in patients with suspected GERD-related chronic laryngitis (RR: 1.28; 95% CI: 0.94–1.74) (128). Another meta-analysis found no significant clinical improvement in the asthma symptoms with PPI treatment compared to the control (129).

PPIs are recommended for the treatment of extraesophageal GERD in patients with typical reflux symptoms or objective evidence of GERD on other diagnostic tests. The recommended therapy duration is 8–12 weeks with dose escalation to twice daily as needed (3). According to the 2025 San Diego Consensus, a 3-month trial of twice-daily PPI therapy, with or without alginate, is sufficient to determine improvement in LPR symptoms (69). Previous evidence indicates that twice-daily PPI therapy provides better acid control and symptom improvement compared to once-daily dosing, with symptom improvement observed in >50% of patients who were unresponsive to once-daily PPIs after 8 weeks (127).

**Recommendation 17:** For patients with refractory GERD, it is necessary to evaluate the optimization of medical treatment, including lifestyle modifications and assessment of patient adherence, before considering dose escalation, switching to another PPI, or adding adjunctive therapies.

*Level of evidence: High*
*Recommendation level: Strong*
*Level of agreement: Strongly agree (82.1%); Agree with some concern (10.7%); Agree with major concerns (7.1%)*

The initial management of refractory GERD should focus on continuing lifestyle modification, assessing treatment adherence, and optimizing PPI therapy. If the patient’s response is inadequate, additional treatment with nocturnal H2-receptor blockers, baclofen, alginates, and topical mucosal protectants may be considered. Endoscopic or surgical anti-reflux interventions may be reserved for selected cases. The 2024 IWGCO consensus recommends against increasing PPI doses, adding LES relaxant inhibitors or bile acid sequestrants in patients with reflux symptoms refractory to PPI therapy for 8 weeks if there is no objective evidence of GERD (24)

*Verifying treatment compliance:* Non-adherence is common in GERD patients (20%–50%) (130) and is influenced by sociocultural factors, limited disease awareness, adverse drug reactions, and suboptimal physician–patient communication (131). Adherence to lifestyle and pharmacological therapy should be verified before treatment escalation.
*Using PPI twice daily:* Twice-daily PPI therapy is more effective than once-daily dosing in maintaining intragastric pH of >4 (132–134). It has also been shown to improve persistent and nocturnal symptoms, accelerate mucosal healing, and reduce relapse in patients with inadequate response to standard dosing (135, 136).
*Switching to another PPI:* Most PPIs are metabolized via CYP2C19, and genetic polymorphisms can lead to variable PPI metabolism, in which rapid metabolizers may have lower serum PPI concentrations and reduced acid-suppressive efficacy (137). Therefore, switching to a less CYP2C19-dependent PPI may improve the treatment response in patients with inadequate response to previous therapy (74).
*H2-receptor antagonists*: H2-receptor antagonists may be used in patients with nocturnal heartburn or sleep disturbances associated with GERD (138–140).
*Alginates and mucosal protective agents*: Combination of PPIs with alginates or mucosal protective agents can be beneficial in patients with refractory GERD. Alginates can be used either as monotherapy in patients with mild-to-moderate GERD or in combination with PPI in partially responsive or unresponsive cases (39).
*Baclofen*: Baclofen is a GABA receptor antagonist, thereby inhibiting the TLESRs. It can be considered for refractory GERD patients, who mainly present with regurgitation symptoms and have a positive SAP on esophageal impedance pH monitoring because of its short-term reduction in the number of reflux events, mean duration of reflux events, and frequency of TLESRs (25, 141, 142). However, it should be initiated at a low dose with gradual titration and used with caution in patients with renal impairment or neuropsychiatric disorders due to potential adverse effects, including dizziness, fatigue, headache, and nausea, particularly with prolonged or high-dose use (142, 143)

**Recommendation 18:** Potassium-competitive acid blockers (P-CABs) could be considered for controlling acid secretion in GERD management.

*Level of evidence: Moderate*
*Recommendation level: Strong*
*Level of agreement: Strongly agree (60.7%); Agree with some concern (32.1%); Agree with major concerns (7.1%)*

P-CABs inhibit gastric acid secretion by competitively blocking the potassium-binding site of H +/K + -adenosine triphosphatase (ATPase). Their stability in acid allows a once-daily dosing regimen and is independent of mealtimes. Notable representatives of P-CABs include vonoprazan, tegoprazan, zastaprazan, fexuprazan, revaprazan, linaprazan, and keverprazan (144). Several systematic reviews and meta-analyses of clinical trials have demonstrated that PPIs and P-CABs achieved similar safety profiles and healing rates, but P-CABs showed superior and faster efficacy in severe EE (145–150). Vonoprazan (Japan) and tegoprazan (South Korea) have been incorporated into the GERD guidelines in Asia (43, 47). The Japanese GERD guidelines (2021) recommended 20-mg vonoprazan as the first-line treatment of severe EE for 4 weeks, followed by maintenance therapy with vonoprazan 10–20 mg or step-down to minimal-dose PPI if the symptoms are controlled. In mild GERD, the efficacy of P-CABs and PPIs may be similar to either 20-mg vonoprazan daily or a standard-dose PPI. Regarding PPI-refractory GERD, 20-mg vonoprazan daily (up to 8 weeks), double-dose PPI, or combination therapy can be considered (43, 47, 144). Further research is needed to evaluate the long-term efficacy, safety, cost-effectiveness, and accessibility of P-CAB.

Although the level of evidence is rated as moderate due to the limited number of large-scale randomized trials and the lack of long-term comparative data, the panel issued a strong recommendation based on the consistent demonstration of clinical benefit, particularly the better speed and level of acid suppression in severe EE and PPI-refractory cases. At present, P-CABs are not yet approved or commercially available in Vietnam, and no local data are available for their use in this setting. As P-CABs become more widely accessible across Asia, further research is needed to evaluate their long-term efficacy, safety, cost-effectiveness, and feasibility for integration into GERD management, including in Vietnamese populations.

**Recommendation 19:** Endoscopic or surgical anti-reflux procedures may be considered for selected patients who have objective evidence of GERD and continue to experience troublesome typical reflux symptoms despite optimized medical therapy.

*Level of evidence: Low*
*Recommendation level: Strong*
*Level of agreement: Strongly agree (64.3%); Agree with some concern (14.3%); Agree with major concerns (10.7%); Disagree with some concern (3.6%); Strongly disagree (7.1%)*

Endoscopic or surgical antireflux interventions should only be considered in patients with a confirmed GERD diagnosis and refractory typical symptoms despite undergoing optimal medical therapy (20). Before interventions, a comprehensive evaluation for other esophageal motility disorders (distal esophageal spasm, EGJ obstruction, achalasia, and absent contractility) is necessary, especially in patients with dysphagia or chest pain (3, 151). The choice of antireflux intervention should be individualized based on the patient’s anatomical abnormalities and preference, surgeons’ expertise, and availability in medical facilities.

As for the surgical intervention, Nissen fundoplication and its modifications, including Toupet, Watson, and Dor fundoplication, are widely applied in clinical practice due to their long-term efficacy in terms of controlling the GERD symptoms and reducing PPI usage compared with both active and maintenance medical therapy, with low short- and long-term complication rates (79, 80, 152). In Vietnam, a study on 34 refractory GERD patients who underwent laparoscopic Nissen fundoplication at Can Tho General Hospital (2023–2025) reported that the procedure was safe and effective. Postoperative outcomes were favorable, with 94.1% achieving good results and 5.9% fair outcomes, with minimal complications (153). Anti-reflux endoscopic interventions include anti-reflux mucosectomy (ARMS), resection and plication (RAP), anti-reflux mucosal ablation (ARMA), anti-reflux mucosectomy band, and transoral incisionless fundoplication (TIF) (154, 155). Meta-analyses and long-term longitudinal studies have reported that endoscopic interventions, such as ARMS and TIF, have low complication rates and are effective for managing refractory GERD with high response and long-term efficacy rates, including reduced AET, improved quality of life, and reduced PPI usage (156–159). However, published data from Vietnam on the application of these emerging endoscopic approaches remains limited.

Although the current level of evidence for endoscopic and surgical anti-reflux interventions is rated as low, a strong recommendation is justified based on the clinical benefits observed across available studies and the extensive clinical expertise of the panel in managing refractory GERD, especially surgical options. In the Vietnamese context, both laparoscopic fundoplication and endoscopic anti-reflux procedures are available at several tertiary referral centers, though access remains limited in rural and lower-resource settings. Cost and availability should therefore be considered when individualizing treatment decisions.

## 5 Overlapping treatment of GERD and other disorders of gut–brain interaction

**Recommendation 20:** In patients with GERD overlapping with other disorders of gut–brain interactions (DGBIs), treatment should include a combination of acid suppression and adjunctive pharmacologic therapies.

*Level of evidence: Moderate*
*Recommendation level: Strong*
*Level of agreement: Strongly agree (75%); Agree with some concern (14.3%); Agree with major concerns (3.6%); Disagree with some concern (3.6%); Strongly disagree (3.6%)*

DGBIs involve multiple mechanisms, including visceral hypersensitivity, increased mucosal permeability, immune dysfunction, and gut–brain axis dysregulation (160). A systematic review reported functional dyspepsia in 41.15% of patients with GERD (95% CI: 29.46–53.93) (77). In Japan (*n* = 2,680), functional dyspepsia and IBS were present in 31.4% and 29.5% of patients with GERD, respectively (161), whereas a Vietnamese study (n = 439) found a GERD–functional dyspepsia overlap at a rate of 51.3% (162).

Given the role of motility disorders, multiple guidelines (Thailand 2018, Korea 2020, VNAGE 2022, BSG, CAG, ACG) recommend PPIs combined with prokinetics, particularly in functional dyspepsia with postprandial distress syndrome (163–167)

Patients with overlapping GERD and DGBIs have more severe symptoms and poorer quality of life compared with those with GERD alone. A cross-sectional study conducted in the United States reported the highest symptom burden in GERD–functional dyspepsia–IBS-C (73.5%), followed by GERD–IBS-C (65.7%) and GERD–functional dyspepsia (53.5%), versus 28.3% for GERD alone (168). A Taiwanese study involving 111 patients with GERD showed that an overlap with functional dyspepsia was associated with poorer mental and physical quality of life, whereas concurrent IBS was linked to worse sleep quality and more severe symptoms of depression (*p* < 0.0001) (169, 170). Consequently, the guidelines recommend tricyclic antidepressants (TCAs) for refractory functional dyspepsia and IBS (3, 163, 166, 167, 171, 172), which are often combined with prokinetics, and initiated at low doses with gradual titration.

Patients with refractory GERD frequently have overlapping disorders of gut–brain interaction (DGBIs). A Vietnamese study in patients with persistent reflux symptoms despite 8 weeks of PPI therapy also reported a high prevalence of anxiety and depression, supporting the importance of evaluating gut–brain interaction and psychological comorbidities in refractory GERD (173). Consequently, several guidelines recommend low-dose tricyclic antidepressants (TCAs) as neuromodulators for selected patients with refractory functional dyspepsia and IBS, particularly when overlapping with GERD (3, 174, 175).

## 6 Conclusion

GERD is a common gastrointestinal disease in Vietnam, and its prevalence has markedly increased in recent years. The VNAGE guideline outlines the recommendations on the diagnosis and management of GERD. Further evidence from local research is warranted to address several critical clinical aspects and support future guideline updates.
