# Supplementary material for: Vietnam Association of Gastroenterology (VNAGE) guideline on the management of gastroesophageal reflux disease
Source: Front Med (Lausanne). 2026 Aug 3;13:1890726. doi: 10.3389/fmed.2026.1890726 (PMC13477947; doi:10.3389/fmed.2026.1890726)
Supplement: Supplementary file 1 [file Supplementary_file_1.docx]

**SUPPLEMENTARY APPENDIX**

**Table S1: PICO questions for the VNAGE GERD guideline**

| **PICO question** | **Statements** |
| --- | --- |
| In patients with suspected GERD who present exclusively with atypical symptoms, does a multidisciplinary evaluation improve diagnostic accuracy and patient outcomes? | 1 |
| In patients with suspected GERD, are clinical questionnaires or an empirical PPI trial, compared with objective diagnostic testing, sufficiently accurate to establish a definitive diagnosis of GERD? | 2 |
| In patients with suspected GERD who have alarm symptoms, persistent symptoms despite an adequate PPI trial, or are being considered for endoscopic or surgical anti-reflux therapy, does upper endoscopy compared with no or delayed endoscopy improve diagnostic yield and clinical management? | 3 |
| Among adults with GERD receiving PPI therapy, do patients with NERD, compared with those with erosive reflux disease, have lower rates of symptom relief and treatment response? What is the prevalence of complications in GERD patients? | 4 |
| In adults with suspected GERD who have persistent symptoms, inadequate response to PPI therapy, normal or minimally abnormal endoscopy, or candidates for anti-reflux intervention, does 24-hour ambulatory esophageal pH monitoring, compared with clinical assessment or endoscopy alone, improve diagnostic accuracy and patient management? | 5 |
| In patients with suspected GERD, what criteria define a definitive diagnosis? | 6 |
| In adults with suspected GERD who have persistent symptoms despite treatment, symptoms suggestive of an esophageal motility disorder, or candidates for endoscopic or surgical anti-reflux procedures, does high-resolution esophageal manometry improve diagnostic accuracy, patient selection, and clinical outcomes? | 7 |
| In adults with suspected GERD, do rapid salivary pepsin detection or esophageal mucosal impedance measurement, compared with established diagnostic methods, improve diagnostic accuracy and clinical decision-making? | 8 |
| In adults presenting with extraesophageal symptoms suggestive of GERD, does multidisciplinary evaluation before initiating an empirical PPI trial improve diagnostic accuracy and patient management? | 9 |
| In patients with persistent GERD symptoms despite medical therapy, what criteria define a diagnosis of refractory GERD? | 10 |
| In patients with GERD, does an individualized treatment approach improve outcomes compared to a standardized treatment protocol? | 11 |
| In patients with GERD, do lifestyle modifications compared with no intervention improve symptom control? | 12 |
| In patients with GERD, does PPI-based therapy improve symptom relief and mucosal healing compared to non-PPI or shorter treatment regimens? | 13, 14, 15 |
| In patients with extraesophageal symptoms, does confirmation of GERD before initiating PPI therapy improve treatment outcomes compared to empirical PPI use? | 16 |
| In patients with refractory GERD, does optimizing existing therapy before considering dose escalation, switching to another PPI, or adding adjunctive therapies improve the outcomes? | 17 |
| In patients with GERD, do potassium-competitive acid blockers provide superior acid control compared to PPIs? | 18 |
| In patients with GERD refractory to medical therapy, do endoscopic or surgical anti-reflux procedures improve symptom outcomes compared to continued pharmacologic management? | 19 |
| In patients with GERD overlapping with functional gastrointestinal disorders, does combination therapy with adjunctive pharmacologic therapy improve symptom control compared to acid suppression alone? | 20 |

**Table S2. Parameters used in 24-hour pH-impedance monitoring [176]**

| **Parameters** | **Definition** | **Normal value** | **Diagnosis of pathological GERD according to Lyon 2018** | **Diagnosis of pathological GERD according to Lyon 2023 (off PPI therapy)** |
| --- | --- | --- | --- | --- |
| Esophageal acid exposure time (AET) - % | The duration of esophageal mucosal exposure to acid is relative to the total monitoring time, indicating the extent to which the esophageal mucosa is exposed to an environment with pH < 4. | < 4% | - >6%: confirmed diagnosis of GERD - 4-6%: inconclusive diagnosis of GERD - < 4%: GERD excluded | - > 6% on 24-hour pH monitoring with/without impedance, or > 6% on ≥ 2 days of wireless pH monitoring: confirmed diagnosis of GERD - 4-6% on 24-hour pH monitoring with/without impedance, or 4-6% on ≥ 2 days of wireless pH monitoring: inconclusive diagnosis of GER - D< 4% on all days of wireless pH monitoring: GERD excluded |
| DeMeester score | A combined score of acid exposure during 24-hour pH-impedance monitoring. | < 14.72 |  |  |
| Post-reflux swallow-induced peristaltic wave (PSPW) - % | A secondary peristaltic wave induced by swallowing after reflux, reflecting the ability to clear refluxate and associated with healing of the esophageal mucosa. | > 61% | ≤ 61%: supports GERD |  |
| Mean nocturnal baseline impedance (MNBI) - Ohm | Mean nocturnal baseline impedance is calculated by averaging impedance during the most stable 10-minute periods (without reflux or swallowing episodes) at 1, 2, and 3 a.m. (when the patient is asleep). | ≥ 2292 Ohm | < 2292 Ohm: supports GERD | < 1500 Ohm: supports GERD |
| Symptom Index (SI) - % | The percentage of symptoms associated with reflux episodes relative to the total number of symptoms during the test period. | < 50% | ≥ 50%: supports GERD | ≥ 50%: supports GERD |
| Symptom Association Probability (SAP) - % | The 24-hour pH-impedance data are divided into small 2-minute data segments. The analysis software evaluates these segments to determine whether the patient has symptoms and whether they are associated with reflux episodes. | ≤ 95% | > 95%: supports GERD | > 95%: supports GERD |

**Table S3. FSSG Questionnaire**

| **Question** | *Never* | *Occasionally* | *Sometimes* | *Often* | *Always* |
| --- | --- | --- | --- | --- | --- |
| Do you get heartburn? | 0 | 1 | 2 | 3 | 4 |
| Does your stomach get bloated? | 0 | 1 | 2 | 3 | 4 |
| Does your stomach ever feel heavy after meals? | 0 | 1 | 2 | 3 | 4 |
| Do you sometimes subconsciously rub your chest with your hand? | 0 | 1 | 2 | 3 | 4 |
| Do you ever feel sick after meals? | 0 | 1 | 2 | 3 | 4 |
| Do you get heartburn after meals? | 0 | 1 | 2 | 3 | 4 |
| Do you have an unusual (e.g. burning) sensation in your throat? | 0 | 1 | 2 | 3 | 4 |
| Do you feel full while eating meals? | 0 | 1 | 2 | 3 | 4 |
| Do some things get stuck when you swallow? | 0 | 1 | 2 | 3 | 4 |
| Do you get bitter liquid (acid) coming up into your throat? | 0 | 1 | 2 | 3 | 4 |
| Do you burp a lot? | 0 | 1 | 2 | 3 | 4 |
| Do you get heartburn if you bend over? | 0 | 1 | 2 | 3 | 4 |

An FSSG score ≥ 8 has diagnostic value for GERD, with a sensitivity of 62% and a specificity of 59% [14].

**Table S4. GerdQ Questionnaire**

|  |  | *Score corresponding to the number of days symptoms occurred during the past week* | | | |
| --- | --- | --- | --- | --- | --- |
|  | **Question** | *0 day* | *1 day* | *2-3 days* | *4-7 days* |
| S+ | How often did you have a burning feeling behind your breastbone (heartburn)? | 0 | 1 | 2 | 3 |
| S+ | How often did you have stomach contents (liquid or food) moving upwards to your throat or mouth (regurgitation)? | 0 | 1 | 2 | 3 |
| S- | How often did you have pain in the centre of the upper stomach? | 3 | 2 | 1 | 0 |
| S- | How often did you have nausea? | 3 | 2 | 1 | 0 |
| I+ | How often did you have difficulty getting a good night's sleep because of your heartburn and/or regurgitation? | 0 | 1 | 2 | 3 |
| I+ | How often did you take additional medication for your heartburn and/or regurgitation, other than what the physician told you to take) (such as Tums, Rolaids, Maalox?) | 0 | 1 | 2 | 3 |

S+: positive symptom; S-: negative symptom; I+: impact.

A GerdQ score ≥ 8 has diagnostic value for GERD, with sensitivity and specificity of 64.6% and 71.4%, respectively [13].

**Table S5. Strength of recommendations for lifestyle and dietary modification measures in GERD management according to international societies**

| **Recommended dietary and lifestyle modification measures** | **Level of evidence** | | | **Strength of recommendation** | | |
| --- | --- | --- | --- | --- | --- | --- |
|  | ACG 2022 [3] | SEA (2021) [39] | Asia-Pacific Consensus (2008) [177] | ACG 2022 [3] | SEA (2021) [39] | Asia-Pacific Consensus (2008) [177] |
| Weight loss | ++ | +++ | ++ | ++ | ++ | ++ |
| Head-of-bed elevation | + | ++ | N/A | + | ++ | N/A |
| Smoking cessation | + | ++ | N/A | + | ++ | N/A |
| Reduce alcohol consumption | N/A | ++ | N/A | N/A | ++ | N/A |
| Dietary modification | + | ++ | N/A | + | ++ | N/A |

*Notes:*

*Level of evidence: High (+++), Moderate (++), Low (+)*

*Strength of recommendation: Strong (++), Conditional/Weak (+)*

*N/A: No information available*

*ACG (American College of Gastroenterology)*

*SEA: Southeast Asian consensus*

**Table S6. Standard doses of PPI medications**

| **PPI medication** | **Standard dose** |
| --- | --- |
| Omeprazol | 20 mg |
| Pantoprazol | 40 mg |
| Lansopraxol | 30 mg |
| Dexlansoprazol | 30mg |
| Esomeprazol | 40 mg |
| Rabeprazol | 20 mg |

The selection of PPI dose and duration of use depends on each individual case. Timing of administration: PPIs should be taken 30-60 minutes before meals to optimize treatment efficacy, except for dexlansoprazole because its effect is not meal-dependent.
